# Comparison of Superovulated Embryo Quality in Simmental Cattle Inseminated with 0 °C-Refrigerated and Liquid Nitrogen-Frozen Semen

**DOI:** 10.3390/biology14060658

**Published:** 2025-06-06

**Authors:** Jie-Ru Wang, Fei Huang, Peng Niu, Hong Cheng, Hui-Min Qu, Xiao-Peng Li, Xue-Yan Wang, Jie Wang, Jia-Jia Suo, Di Fang, Qing-Hua Gao

**Affiliations:** 1 College of Animal Science and Technology, Tarim University, Alar 843300, China; 107572021314@stumail.taru.edu.cn (J.-R.W.); 107572022315@stumail.taru.edu.cn (F.H.); 10757231088@stumail.taru.edu.cn (H.-M.Q.); miexiaochi@163.com (X.-P.L.); wangxueyan1016@163.com (X.-Y.W.); 120240063@taru.edu.cn (J.W.); 107572021317@stumail.taru.edu.cn (J.-J.S.); 120230093@taru.edu.cn (D.F.); 2 College of Life Science and Technology, Tarim University, Alar 843300, China; 107572023420@stumail.taru.edu.cn (P.N.); 107572024416@stumail.taru.edu.cn (H.C.); 3 Key Laboratory of Tarim Animal Husbandry Science and Technology, Xinjiang Production & Construction Corps, Alar 843300, China

**Keywords:** 0 °C-refrigerated semen, liquid nitrogen-frozen semen, superovulation, in vivo embryos, embryo quality

## Abstract

Semen quality plays a crucial role in bovine in vivo embryo production. This study aimed to compare the effects of 0 °C-refrigerated semen and liquid nitrogen-frozen semen on embryo quality in Simmental cattle. Semen collected from five bulls was equally divided into two groups: one diluted with a 0 °C refrigeration solution and stored at 0 °C, and the other diluted with a cryopreservation solution and stored in liquid nitrogen for 24 h. We evaluated sperm motility, progressive motility (assessed via a computer-assisted sperm analyzer), acrosome integrity, and plasma membrane integrity in both groups. Fifty superovulated Simmental cows were artificially inseminated with semen from both groups. Embryos were non-surgically flushed on day seven, followed by BrdU proliferation staining and TUNEL apoptosis staining. Proliferation and apoptosis levels were quantified using marker genes. Results showed that 0 °C-refrigerated semen exhibited significantly higher sperm motility, progressive motility, acrosome integrity, and plasma membrane integrity compared to liquid nitrogen-frozen semen (*p* < 0.05). While total embryo numbers showed no significant difference between groups (*p* ≥ 0.05), embryos from 0 °C-refrigerated semen contained significantly more proliferative cells (*p* < 0.05) and fewer apoptotic cells (*p* < 0.05) than those from frozen semen. These findings demonstrate that 0 °C-refrigerated semen outperforms liquid nitrogen-frozen semen in both sperm quality parameters and resultant embryo quality.

## Simple Summary

Semen quality plays a crucial role in bovine embryo production in vivo; however, the effects of 0 °C-refrigerated semen compared to liquid nitrogen-frozen semen on the resulting embryo quality have not been extensively studied. This study aims to compare the effects of 0 °C-refrigerated semen and liquid nitrogen-frozen semen on embryo quality. The findings indicate that 0 °C-refrigerated semen demonstrates significantly superior sperm motility, progressive motility, acrosome integrity, and plasma membrane integrity when compared to liquid nitrogen-frozen semen. Furthermore, the embryo quality derived from 0 °C-refrigerated semen is significantly higher than that produced from liquid nitrogen-frozen semen.

## 1. Introduction

Semen quality plays a crucial role in embryo production. High-quality sperm that fertilize oocytes are essential for generating viable embryos [1,2,3]. In recent years, artificial insemination technology has gained widespread adoption, primarily utilizing liquid nitrogen-frozen semen. This method offers several advantages, including ease of use and extended preservation capabilities [4,5]. However, the production of liquid nitrogen-frozen semen necessitates cryogenic freezing treatment. Compared to non-frozen sperm, frozen sperm exhibit deficiencies in protein content, functionality, oxidative stress damage mitigation, motility, and DNA integrity [6,7,8]. The vitality of semen significantly decreases after being frozen in liquid nitrogen, leading to a reduction in embryo quality. Therefore, enhancing embryo quality cannot be achieved through the use of liquid nitrogen-frozen semen [9]. Despite the long-term preservation benefits of liquid nitrogen-frozen semen, the damage incurred during the freezing process is irreversible [10,11].

Semen refrigerated at 0 °C within its preservation window causes minimal damage to sperm. Studies indicate that ram semen refrigerated at 0 °C for 12 days remains viable for artificial insemination [12,13]. Reports also indicate that Simmental bull semen refrigerated at 0 °C for 9 days maintains sperm motility above 50% [14], demonstrating that refrigeration at this temperature is fully feasible for cattle applications. Due to the poor tolerance of ram semen to liquid nitrogen freezing [15], research on 0 °C-refrigerated ram semen significantly outpaces that of bovine semen. Since research on 0 °C-refrigerated bovine semen is relatively scarce, studies on embryos derived from such refrigerated bovine semen are even less documented. In the artificial insemination of superovulated cattle [16], the synchronization of donor cattle allows experimenters to precisely determine estrus timing and insemination timing. Therefore, in terms of both sperm quality and cost-effectiveness, the use of 0 °C-refrigerated bovine semen proves to be far superior to liquid nitrogen-frozen semen.

This study compares sperm quality and embryo development using bovine semen refrigerated at 0 °C versus semen cryopreserved in liquid nitrogen. We employed a self-formulated diluent for bovine semen refrigerated at 0 °C (Patent No. ZL2007100181613, IPC Classification No. CN100581553C) [16]. Sperm motility, progressive motility, acrosome integrity, and plasma membrane integrity were assessed between Simmental cattle liquid nitrogen-frozen semen and 0 °C-refrigerated semen, as was the quality of embryos generated from both methods. Our findings demonstrate that the embryo quality derived from 0 °C-refrigerated bovine semen is significantly superior to that produced by liquid nitrogen-frozen bovine sperm.

## 2. Materials and Methods

### 2.1. Ethics Statement

All animal experiments were conducted following the “Regulations and Guidelines for the Management of Experimental Animals” established by the Ministry of Science and Technology (Beijing, China, 2020 revision). This study was approved by the Institutional Animal Care and Use Committee of Tarim University, Xinjiang, China (protocol code DWBH20220101; approval date: 1 January 2022).

### 2.2. Experimental Procedure

**Figure 1:**
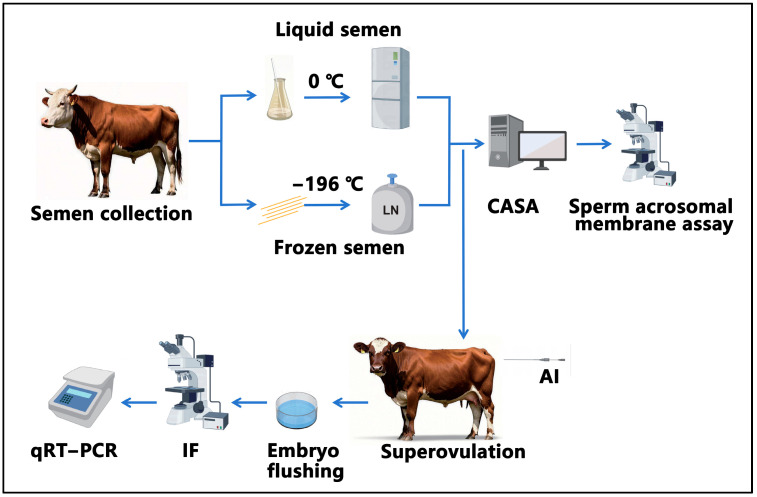
Experimental flowchart.

The study design is shown in Figure 1. First, we collected semen from five bulls. The collected semen was processed and then preserved in either liquid nitrogen or at 0 °C for 24 h. Subsequently, computer-assisted analysis and assessment of the integrity of the acrosomal membrane were conducted. Following this, artificial insemination was performed. On the 7th day after artificial insemination, embryo flushing was carried out, followed by the immunofluorescence observation of the embryos and detection of genes related to proliferation and apoptosis.

### 2.3. Semen Collection and Processing

In this experiment, semen was collected from five breeding bulls using the artificial vagina method at the Dingxin Breeding Bull Farm in Xinhe County, Xinjiang, China. Following collection, an initial motility assessment was performed through a subjective visual evaluation of sperm motility. The semen from each bull was subsequently divided into two aliquots, and one portion was diluted with a cryopreservation extender (020996, Optidyl, Kasu Biotechnology Co., Ltd., Shanghai, China). After dilution, the semen was loaded into straws (Cat. No.: BOU023892) and processed into frozen semen using a semen freezing machine (DT-LX0, Detou Temmoku, Hefei, China). The cryopreservation procedure involved placing the tubed semen samples into a 4 °C semen freezing machine for a two-hour equilibration period. Subsequently, liquid nitrogen vapor was employed for rapid freezing, reducing the temperature from 4 °C to −120 °C within 7 min, and maintaining it at −120 °C for 10 min. The semen was then swiftly transferred into liquid nitrogen for cryogenic storage. The other portion was diluted with a laboratory-formulated refrigeration extender at 0 °C and stored at this temperature. All pharmaceutical ingredients required for the diluent formulation were supplied by Biotopped Biotechnology Co., Ltd., located in Beijing, China, in the following specific ratios: 5.25 g of Tris (hydroxymethyl) aminomethane (Cat. No.: T6061T), 2.02 g of citric acid (Cat. No.: BZ54859), 3.22 g of fructose (Cat. No.: F6100D), 50 mL of fresh egg yolk, 600,000 IU of Penicillin-Streptomycin Solution(Cat. No.: Top0016S), and 200 mL of sterile water. These components were gently stirred for 30 min to ensure homogeneity. The post-dilution sperm concentration was maintained at 40 million/mL in all samples.

### 2.4. Thawing of Semen

For the thawing of frozen semen, it was removed from liquid nitrogen and rapidly placed in a 38 °C water bath while being gently shaken for 30 s. Subsequently, the semen was collected into centrifuge tubes for further use. For semen that was refrigerated at 0 °C, the procedure involved taking it out, placing it in a 38 °C water bath with gentle shaking for 30 s, and then collecting it into centrifuge tubes for application.

### 2.5. Computer-Assisted Analysis

After 24 h of semen preservation, the sperm motility parameters were analyzed using the Computer-Assisted Sperm Analysis (CASA) system (ML-500JZ, Marang, Shanghai, China) in conjunction with an inverted microscope (TS100-F, Nikon, Tokyo, Japan). Prior to analysis, sperm samples underwent washing via centrifugation. An equal volume of phosphate-buffered saline (PBS) (P1020, Solarbio Science & Technology Co., Ltd., Beijing, China) was added to the semen in a centrifuge tube, gently mixed, and centrifuged at 300 rpm for 3 min. The supernatant was discarded, and this washing process was repeated twice. A 10 μL aliquot of semen was carefully injected into a 20 μm depth sperm counting plate (ML-CASA20-4, Mailang, Ningbo, China), ensuring that no air bubbles formed. The slide was then placed on a microscope stage preheated to 37 °C. The illumination intensity of the microscope was adjusted for optimal visualization on the monitor. Sperm trajectories were captured at 60 Hz (0.5 s, 30 frames) to measure sperm concentration, motile sperm ratio, and CASA parameters, including three velocity metrics: curvilinear velocity (VCL), straight-line velocity (VSL), and average path velocity (VAP). Observations were conducted from at least five randomly selected fields, with triplicate repeats for each sample.

### 2.6. Sperm Acrosome and Plasma Membrane Integrity Detection

The FITC-PNA/DAPI staining method (MP6327; MX4208; Maokang Biotechnology Co., Ltd., Shanghai, China) was employed in this study. Initially, 20 μL of sperm from various groups was placed on a glass slide, air-dried, and fixed with a 2% paraformaldehyde solution (MM1515; Maokang Biotechnology Co., Ltd., Shanghai, China) for 10 min, followed by air-drying. Subsequently, 2 mL of PBS was added to the slide and incubated for 10 min, being repeated twice to eliminate residual paraformaldehyde. Next, 30 μL of the FITC-PNA working solution was evenly applied to cover the sperm sample, incubated in darkness at 37 °C for 30 min, and washed three times with PBS to remove excess dye. Following this, 30 μL of DAPI staining solution was uniformly applied to the slide, incubated in darkness at room temperature for 5–10 min, and immediately observed and imaged under a fluorescence microscope (Nikon Ti2-U), ensuring that light exposure was strictly avoided throughout the process. Sperm exhibiting complete bright green fluorescence in the anterior acrosome were classified as intact acrosome sperm, whereas those with incomplete or absent fluorescence were considered acrosome-damaged. Imaging was conducted in complete darkness. The acrosome integrity rate was calculated as follows: Acrosome Integrity Rate (%) = (Number of intact acrosome sperm/Total sperm count) × 100%. For plasma membrane integrity assessment, the SYBR 14/PI staining method (MX4239; MX4205; Maokang Biotechnology Co., Ltd., Shanghai, China) was utilized following the same protocol as the FITC-PNA/DAPI staining. Sperm exhibiting green fluorescence were identified as viable, while red fluorescence indicated a loss of membrane integrity. The membrane integrity rate was calculated as follows: Membrane Integrity Rate (%) = (Number of membrane-intact sperm/Total sperm count) × 100%. Sperm were observed across at least five randomly selected fields, and each sample was analyzed in triplicate.

### 2.7. Superovulation Treatment, Artificial Insemination, and Non-Surgical Embryo Flushing

**Figure 2:**
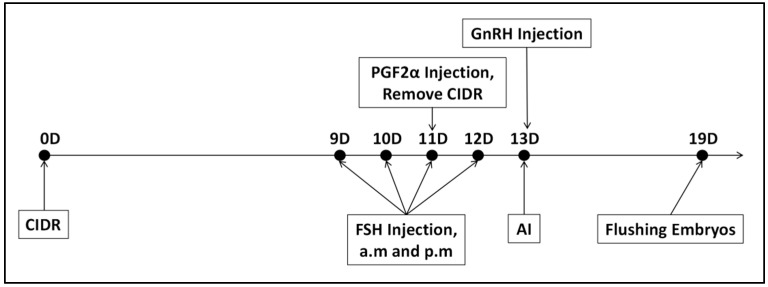
Flowchart of the superovulation protocol for donor cows.

Superovulation Treatment: This study employed the CIDR + FSH + PGF2α + GnRH protocol for superovulation in donor cows [17]. Follicle-stimulating hormone (FSH) plays a crucial role in stimulating the ovaries to release multiple oocytes within a single cycle. The experimental protocol is depicted in Figure 2. On Day 0, a Controlled Internal Drug Release (CIDR) device, containing progesterone, was inserted intravaginally. From Day 9 to Day 12, FSH preparation (700 IU/vial, 20 mL/vial, Vetoquinol™, Brisbane, Australia) was administered intramuscularly twice daily at 12 h intervals, following a decreasing dosage regimen: Day 9: 140 IU (2 doses); Day 10: 105 IU (2 doses); Day 11: 70 IU (2 doses); Day 12: 35 IU (2 doses). On Day 11, 30 μg of PGF2α (10 vials/box, 0.2 mg/vial, NSHF™, Beijing, China) was injected in the morning following FSH administration, and the CIDR was removed in the afternoon. On Day 13, 30 μg of GnRH (100 μg/vial, NSHF™, China) was injected, after which follicular development was monitored through rectal examination and B-ultrasound.

Artificial insemination was conducted 8 to 12 h after the cows exhibited estrus. A total of 25 cows received semen refrigerated at 0 °C, while another 25 cows were inseminated with liquid nitrogen-frozen semen. Insemination was repeated every 12 h, with a third insemination performed if estrus persisted. The first artificial insemination occurred 24 h post-sperm cryopreservation, the second at 36 h, and the third at 48 h. On Day 19, rectal and B-ultrasound examinations were employed to detect and record the number of corpora lutea formed on the ovaries, followed by non-surgical embryo collection.

Non-surgical embryo flushing was performed as follows: Initially, the donor cow received 150 μg of lidocaine hydrochloride for local anesthesia. After ensuring proper disinfection, a dilator rod was inserted into the cervix, followed by the insertion of an embryo collection catheter into the uterus. The catheter balloon was inflated to occlude the uterine horn, depending on its size. A syringe-type embryo flushing catheter was utilized to draw phosphate-buffered saline (PBS) using a 50 mL syringe. PBS was infused through the catheter into the uterine horn, which was gently massaged via rectal manipulation, and the PBS was subsequently aspirated into an embryo collection cup. Each uterine horn was flushed with 500 mL of PBS. The collected embryos were evaluated under a microscope and classified into four grades (1, 2, 3, and 4) based on morphology and developmental stage. In this study, Grade 1 and 2 embryos were defined as viable embryos. Grade 1 embryos: The embryos have a symmetrical and spherical mass with individual blastomeres that are uniform in size, color, and density. Irregularities should be relatively minor, and at least 85% of the cellular material should be an intact, viable embryonic mass. The zona pellucida should be smooth. Grade 2 embryos: These embryos have moderate irregularities in the overall shape of the embryonic mass or in size, color, and density of individual cells. At least 50% of the embryonic mass should be intact [18].

### 2.8. Detection of Proliferation and Apoptosis in Embryos

Both proliferation and apoptosis assays were conducted using Grade 1 Blastocyst. Proliferation rates in the blastocyst were assessed via the BrdU assay. The collected embryos were washed three times with PBS buffer and fixed in a 4% paraformaldehyde solution for 30 min. After being transferred to PBS three times, the embryos were treated with 0.5% acid Tyrode’s solution for 5 min. Following three additional washes with PBS, the embryos were transferred to 0.5% Triton X-100 for 30 min and washed thrice with PBS. Subsequently, the embryos were incubated overnight with BrdU antibody (1:100 dilution; B100-1 μL, Sigma, Burlington, MA, USA) at 4 °C. After three washes with PBS, embryos were incubated with FITC-conjugated goat anti-rabbit IgG (1:1000; ab6939, Abcam, Shanghai, China) at room temperature for 2 h. Following thorough PBS washes, embryos were counterstained with DAPI for 5 min at room temperature. After three additional PBS washes, individual embryos were selected and placed on glass slides with 2 μL of anti-fluorescence quenching mounting medium (P0131; Beyotime Biotechnology, Shanghai, China). Cell proliferation rates were analyzed using a confocal laser scanning microscope (FV1000, Olympus Fluoview system, Olympus Corp, Japan). The proliferation index for each embryo was calculated by dividing the number of BrdU-positive nuclei by the total number of nuclei in the embryo. To assess apoptosis levels in embryonic, embryos were washed three times in PBS buffer and fixed in 4% paraformaldehyde for 30 min. After three PBS washes, embryos were treated with 0.5% acid Tyrode’s solution for 5 min, followed by three PBS washes and a 30 min incubation in 0.5% Triton X-100. Permeabilized embryos were incubated overnight with TUNEL solution in the dark at 4 °C. Stained embryos were subsequently washed and incubated with DAPI for 5 min. After three PBS washes, individual embryos were selected and mounted on slides with 2 μL of anti-fluorescence quenching medium. Imaging was conducted using the confocal laser scanning microscope (FV1000, Olympus Fluoview system). The average percentage of TUNEL-positive nuclei per embryo was determined by dividing the number of positive nuclei by the total cell count (stained with DAPI). At least five randomly selected blastocysts were observed, and each sample was analyzed in triplicate.

### 2.9. Embryo RNA Extraction and Detection of Proliferation-and Apoptosis-Related Genes

cDNA was reverse-transcribed and amplified from single blastocyst using the SMARTer Amplification Kit (634859, Takara Biomedical Technology, Beijing, China). The relative mRNA transcript levels of all tested genes were analyzed via quantitative reverse transcription polymerase chain reaction (qRT-PCR). Proliferation-related genes, including Oct4 and Sox2, were detected, while apoptosis-related genes such as Caspase3 and Bax were also examined. The threshold cycle (Ct) values of all tested genes were normalized to the Ct value of GAPDH, with three replicates established for each sample. The relative expression levels of genes were calculated using the 2^−ΔΔCt^ method. PCR amplification was performed under the following conditions: initial denaturation at 94 °C for 5 min, followed by 40 cycles consisting of 30 s at 94 °C, 30 s at 59 °C, and 30 s at 72 °C. GAPDH served as the reference gene in qRT-PCR, with the average expression level of GAPDH representing the expression level of the reference gene. Five independent experiments were conducted for gene expression analysis, each repeated 3 times. The primers used for qRT-PCR are listed in Table 1.

### 2.10. Statistical Analysis

Statistical graphs were generated using GraphPad Prism software version 8.0 (GraphPad Software, Inc., San Diego, CA, USA). Data are presented as mean ± standard deviation, based on a minimum of three independent experiments. Variables were compared between different groups using Student’s *t*-test (two-tailed). For multiple comparisons, analysis of variance (ANOVA) was employed, followed by pairwise comparisons using the Tukey honest significant difference (Tukey HSD) test. The fluorescence intensity of each image was quantified using ImageJ software (version 1.8.0). A *p*-value of less than 0.05 was considered statistically significant.

## 3. Results

### 3.1. Computer-Assisted Analysis Results

**Figure 3:**
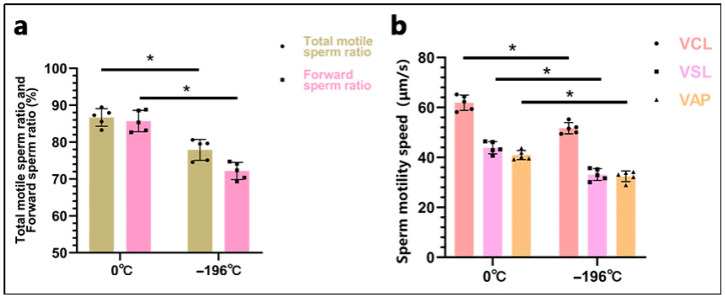
Results of computer-assisted sperm analysis. In the figure. (**a**), Displays the proportion of total motile sperm and progressively motile sperm. (**b**), Displays the sperm motility parameters, including VCL, VSL, and VAP. “0 °C” denotes the 0 °C-refrigerated sperm group, and “−196 °C” represents the liquid nitrogen-frozen sperm group. The asterisk (*) indicates significant differences between the two groups (*p* < 0.05).

Through computer-assisted analysis, we found that the total motile sperm percentage of 0 °C-refrigerated sperm (86.71 ± 2.37) was significantly higher than that of liquid nitrogen-frozen sperm (77.89 ± 2.81) (*p* < 0.05). Similarly, the percentage of progressively motile sperm in 0 °C-refrigerated sperm (84.52 ± 2.90) was significantly greater than that of liquid nitrogen-frozen sperm (72.18 ± 2.31) (*p* < 0.05) (Figure 3a). Additionally, the values for VCL, VSL, and VAP of 0 °C-refrigerated sperm (61.87 ± 3.05, 43.88 ± 2.42, 40.93 ± 1.84) were all significantly higher than those of liquid nitrogen-frozen sperm (51.68 ± 2.27, 33.12 ± 2.37, 32.43 ± 2.11) (*p* < 0.05) (Figure 3b).

### 3.2. Detection of Sperm Acrosome and Plasma Membrane Integrity

**Figure 4:**
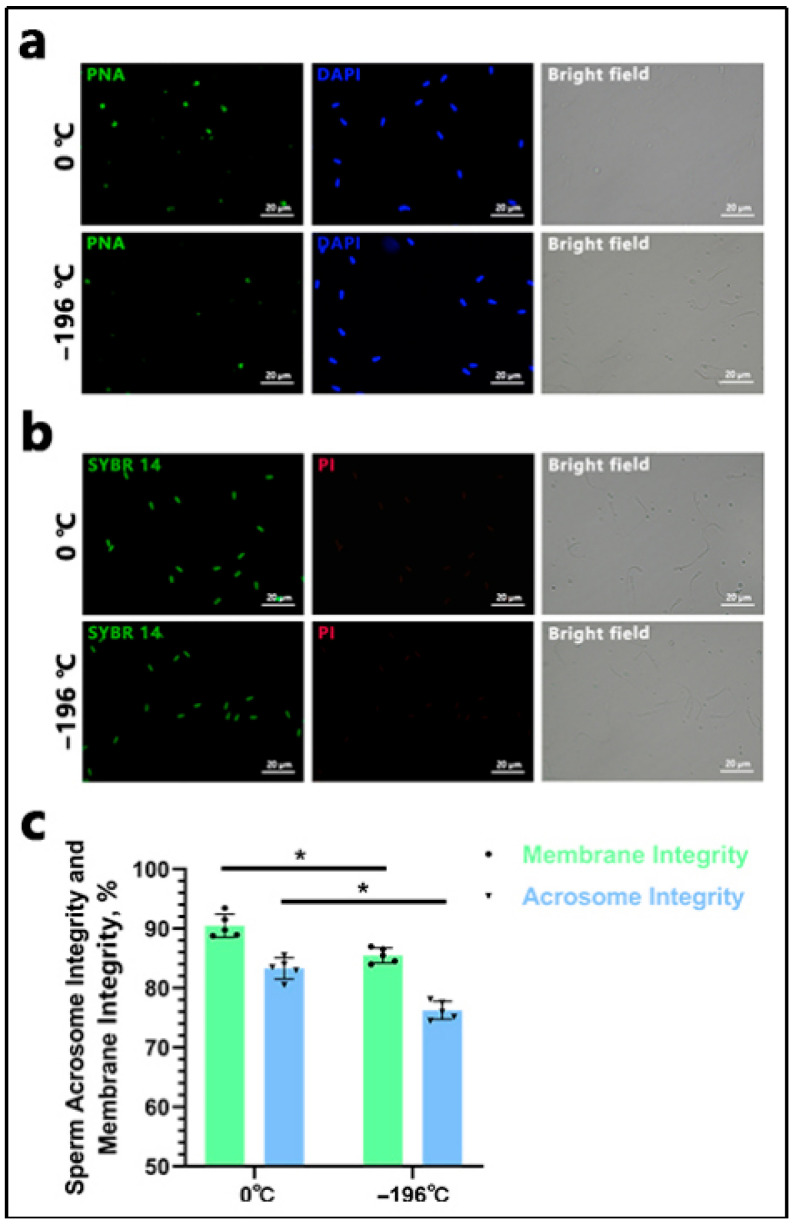
Results of sperm acrosome and plasma membrane integrity detection. In (**a**), “0 °C” denotes the 0 °C-refrigerated sperm group, and “−196 °C” represents the liquid nitrogen-frozen sperm group (hereinafter the same). Green fluorescence indicates acrosome-intact sperm stained with FITC-PNA, while blue fluorescence represents DAPI staining. The scale bar is 20 μm. In (**b**), green fluorescence indicates viable sperm stained with SYBR 14, and red fluorescence represents PI staining (membrane-disrupted sperm). The scale bar is 20 μm. In (**c**), the asterisk (*) indicates significant differences between the two groups (*p* < 0.05).

The sperm acrosome was stained with green fluorescence (Figure 4a). The integrity of the plasma membrane was assessed (Figure 4b). Statistical analysis revealed that the acrosome integrity rate of sperm refrigerated at 0 °C (83.29 ± 1.82) was significantly higher than that of sperm frozen in liquid nitrogen (76.24 ± 1.52) (*p* < 0.05). Similarly, the plasma membrane integrity rate of sperm refrigerated at 0 °C (90.48 ± 1.97) was significantly greater than that of sperm frozen in liquid nitrogen (85.48 ± 1.27) (*p* < 0.05) (Figure 4c).

### 3.3. Embryo Recovery Results

**Figure 5:**
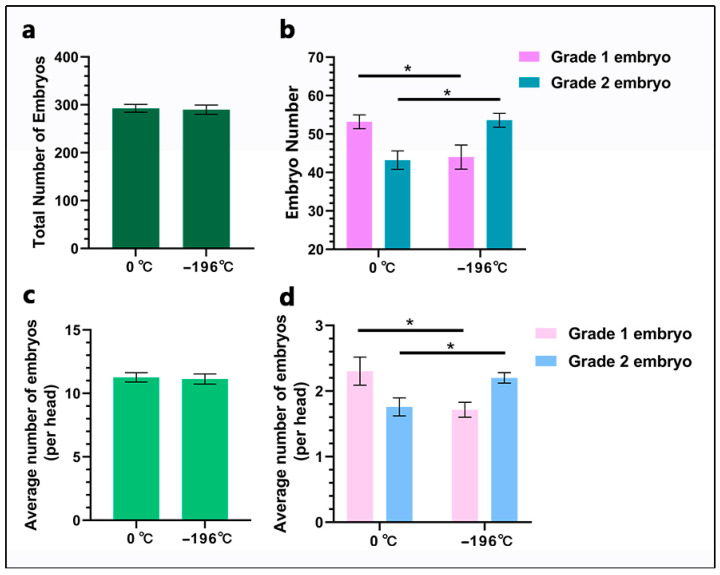
Embryo recovery results. (**a**), Shows the total number of embryos. (**b**), Presents the counts of grade 1 and grade 2 embryos. (**c**), Displays the number of embryos per cow. (**d**), Illustrates the quantity of grade 1 and grade 2 embryos per cow. Unmarked data in the figure indicate no significant difference (*p* ≥ 0.05), while the asterisk (*) denotes a significant difference between the two groups (*p* < 0.05).

The results of non-surgical embryo recovery are illustrated in Figure 5. Total embryo counts showed no significant divergence between the 0 °C cohort (292.6 ± 3.7) and the −196 °C group (289.8 ± 4.3; *p* ≥ 0.05; Figure 5a). Notably, the 0 °C group yielded a substantially greater number of Grade 1 embryos (53.20 ± 1.78, 54.2% of total) compared to the −196 °C group (44.00 ± 3.16, 45.2% of total; *p* < 0.05). In Grade 2 embryos, the opposite trend was observed: the 0 °C group yielded significantly fewer embryos (43.20 ± 2.38, 44.3% of total) compared to the −196 °C group (53.60 ± 1.81, 54.5% of total; *p* < 0.05; Figure 5b). The mean embryo count per individual cow showed no significant difference between groups: 11.71 ± 1.35 (0 °C) versus 11.54 ± 1.46 (−196 °C; *p* ≥ 0.05; Figure 5c). Embryo quality exhibited pronounced contrasts: the 0 °C group displayed higher individual cow counts of Grade 1 embryos (2.13 ± 0.35 vs. 1.76 ± 0.26 in the −196 °C group; *p* < 0.05) while demonstrating reduced individual cow counts of Grade 2 embryos (1.73 ± 0.18 vs. 2.14 ± 0.22 in the −196 °C group; *p* < 0.05; Figure 5d).

### 3.4. Fluorescence Staining Detection of Intracellular Proliferation and Apoptosis in Embryos

**Figure 6:**
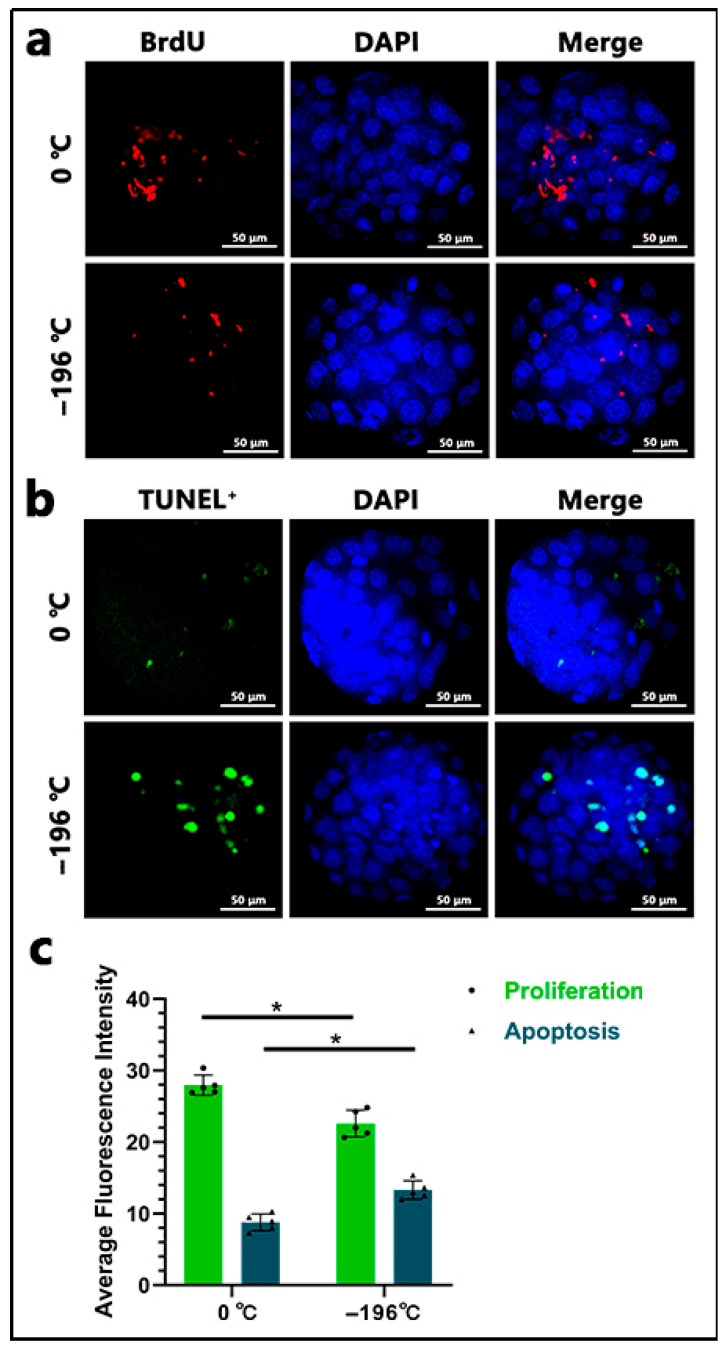
Fluorescence staining results of intracellular proliferation and apoptosis in embryos. In (**a**), red fluorescence indicates BrdU-stained proliferating cells within the embryo, and blue fluorescence represents DAPI staining; the scale bar is 50 μm. In (**b**), green fluorescence indicates TUNEL-stained apoptotic cells, and blue fluorescence represents DAPI staining; the scale bar is 50 μm. In (**c**), the asterisk (*) denotes a significant difference between the two groups (*p* < 0.05).

Proliferating cells within the embryos were stained red using BrdU (Figure 6a), while apoptotic cells were stained green using TUNEL (Figure 6b). Statistical analysis indicated that the fluorescence intensity of proliferating cells in the 0 °C group (27.96 ± 1.40) was significantly higher than that in the −196 °C group (22.59 ± 1.85) (*p* < 0.05). Conversely, the fluorescence intensity of apoptotic cells in the 0 °C group (8.79 ± 1.16) was significantly lower than that in the −196 °C group (13.30 ± 1.29) (*p* < 0.05) (Figure 6c).

### 3.5. Detection of Genes Related to Embryonic Proliferation and Apoptosis

**Figure 7:**
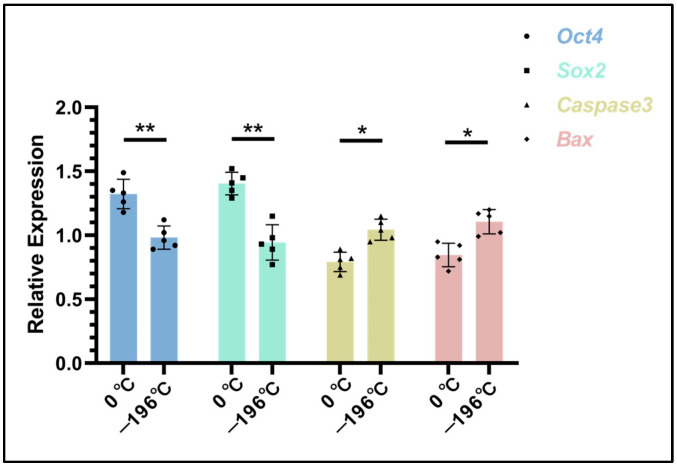
Test results of genes related to embryonic proliferation and apoptosis. In the figure, the asterisk (*) indicates a significant difference between the two groups (*p* < 0.05), and the double asterisk (**) denotes an extremely significant difference (*p* < 0.01).

The analysis of the expression levels of proliferation-related genes (Oct4, Sox2) and apoptosis-related genes (Caspase3, Bax) in embryonic cells (Figure 7) revealed the following findings: The expression levels of Oct4 and Sox2 in the 0 °C group (1.32 ± 0.11, 1.40 ± 0.08) were significantly higher than those in the −196 °C group (0.98 ± 0.09, 0.94 ± 0.13) (*p* < 0.01). In contrast, the expression levels of Caspase3 and Bax in the 0 °C group (0.79 ± 0.07, 0.84 ± 0.09) were significantly lower than those in the −196 °C group (1.04 ± 0.08, 1.10 ± 0.94) (*p* < 0.05).

## 4. Discussion

Previous studies have demonstrated that bull sperm refrigerated at 0 °C exhibits superior parameters, including viability, acrosome integrity, and membrane integrity, compared to sperm frozen in liquid nitrogen, with the exception of storage duration [19,20]. However, to date, no studies have investigated the quality of embryos produced from these two types of sperm. In this study, we compared two semen preservation methods: refrigeration at 0 °C and liquid nitrogen freezing. Subsequent analyses evaluated post-thaw sperm viability, progressive motility, acrosome integrity, and membrane integrity. Additionally, we performed artificial insemination in donor cattle and conducted proliferation/apoptosis detection in the resulting embryos. Our findings indicate that the 0 °C-refrigerated group exhibited significantly better outcomes than the liquid nitrogen-frozen group across all evaluated parameters: sperm viability, progressive motility, acrosome integrity, membrane integrity, and resultant embryo quality.

The quality of semen not only influences fertilization outcomes but also affects the development of early embryos. The use of 0 °C-refrigerated semen challenges the traditional preference for frozen semen in bovine in vivo embryo production [21]. For superovulation and timed artificial insemination protocols, 0 °C-refrigerated semen offers a superior alternative [22]. By refrigerating semen at 0 °C, sperm are protected from damage associated with liquid nitrogen freezing [23], thereby preserving their overall integrity and maximizing embryo quality. Notably, although there was no significant difference in total embryo yield between the two groups, the number of Grade 1 embryos produced from 0 °C-refrigerated semen was significantly higher than that generated from liquid nitrogen-frozen sperm. This finding indicates that the methods of refrigeration and freezing of sperm impact the post-fertilization developmental competence of embryos. Our fluorescence staining experiments (focused on proliferation and apoptosis) and marker gene detection assays consistently supported this conclusion.

In this study, sperm refrigerated at 0 °C exhibited significantly superior performance across all metrics compared to sperm frozen in liquid nitrogen. This improvement can be attributed to two primary factors: first, the avoidance of damage associated with liquid nitrogen freezing in the 0 °C refrigeration protocol; and second, the potential optimization of the extender solution, particularly through enhanced formulations of energy substrates that mitigate chilling-induced injury. While similar extender solutions have been utilized for sheep semen [24,25,26], this study extends their application to bovine reproduction, thereby addressing a critical gap in conventional methodologies that have traditionally prioritized long-term preservation over sperm quality. The feasibility of 0 °C refrigeration aligns well with the synchronization of superovulation schedules [27,28]. In this context, semen is utilized within days post-collection without the need for liquid nitrogen cryopreservation, thus preserving sperm quality while simultaneously enhancing the developmental quality of embryos post-fertilization. With the ongoing intensification of large-scale farming in beef and dairy cattle, estrus synchronization has become increasingly important for management, facilitating fixed-time artificial insemination. The use of liquid semen can significantly improve reproductive performance in cattle. The subjects of this study were Simmental beef cattle. Given the potential differences in semen quality between beef cattle and dairy cattle, future research should consider the long-term preservation of liquid semen from beef cattle. This approach can avoid cryogenic damage caused by liquid nitrogen and enable long-term storage, thereby establishing new methods for semen preservation.

## 5. Conclusions

This study demonstrates that sperm refrigerated at 0 °C exhibits significantly superior motility, progressive motility, acrosome integrity, and plasma membrane integrity compared to sperm frozen in liquid nitrogen. Furthermore, the quality of embryos derived from 0 °C-refrigerated sperm is also significantly better than that from liquid nitrogen-frozen sperm, thereby validating the feasibility of using 0 °C-refrigerated sperm in superovulation-timed artificial insemination.

## Figures and Tables

**Table 1:** List of qRT-PCR primers.

Gene Name	Sequence	Annealing Temperature	Product Length
*Oct4*	F: GGGCAAACGATCAAGCAGTG	60 °C	172 bp
	R: CTCAGGGAATGGGACCGAAG		
*Sox2*	F:AGAGTGTTTGCAAAAGGGGGA	60 °C	147 bp
	R:CGCCGCCGATGATTGTTATT		
*Caspase3*	F: AGCGTCGTAGCTGAACGTAAA	59 °C	247 bp
	R: CTGCATCCACGTCTGTACCA		
*Bax*	F:ACAGGGGCCCTTTTGCTTCA	60 °C	112 bp
	R:TCTCGGGGAGAGTCTGTGTC		
*GAPDH*	F:CTGCCCGTTCGACAGATAGC	60 °C	262 bp
	R:TGATGACGAGCTTCCCGTTC		

## Data Availability

The original contributions presented in the study are included in the article; further inquiries can be directed to the corresponding authors.
